# The Ecological Dynamics Framework: An Innovative Approach to Performance in Extreme Environments: A Narrative Review

**DOI:** 10.3390/ijerph19052753

**Published:** 2022-02-27

**Authors:** Ludovic Seifert, Guillaume Hacques, John Komar

**Affiliations:** 1 Faculty of Sport Sciences, University of Rouen Normandy, CETAPS EA3832, 76821 Mont Saint Aignan, France; guillaume.hacques@univ-rouen.fr; 2 Institut Universitaire de France (IUF), 75231 Paris, France; 3 Physical Education and Sport Sciences, National Institute of Education, Nangyang University of Singapore, Singapore 637616, Singapore; john.komar@nie.edu.sg

**Keywords:** perception–action coupling, complex system, movement variability, motor control and learning, climbing

## Abstract

(1) Background: Uncertainty in extreme sports performance environments, such as climbing, provides considerable psycho-emotional and physiological demands, notably due to the many different environments in which climbing can be performed. This variety of environments, conditions of practice and engagement would challenge the acquisition of perceptual-motor skills; (2) Methods: To better understand how perceptual-motor skills are controlled and acquired in climbing, we proposed a narrative review anchored in the ecological dynamics theoretical framework and showed how this theoretical framework would support a nonlinear pedagogy to skill acquisition and to design safe learning and training situations that are representative of extreme performance contexts; (3) Results: We explained three theoretical pillars and we provide examples for design intervention following nonlinear pedagogy, notably (i) to set a constraint-led approach (in particular task constraint), (ii) to implement conditions of practice (constant vs. variable, imposed vs. self-controlled), (iii) to promote adaptive and creative behavioral variability during practice; (4) Conclusions: The challenge for the extreme sport practitioner is how to set up conditions of practice for efficient exploration in a manner that manages the dangers of performing in uncertain environments. Representing uncertainty within the relative safety of indoor settings may be one approach for preparing climbers for performance in extreme environments.

## 1. Introduction

Uncertainty in extreme sports environments, such as climbing, provides considerable psycho-emotional and physiological demands, notably due to the many different environments in which climbing can be performed. Indeed, climbing can be performed indoor and outdoor, for differing heights, altitudes, surfaces (rock, snow, ice or mixed), tools as support (e.g., ice axes, crampons), protection and engagement (with or without bolts, solo, top-rope or on-sight). This variety of environments, conditions of practice and engagement would challenge the acquisition of perceptual-motor skills. To better understand how perceptual-motor skills are controlled and acquired, an ecological dynamics theoretical framework is proposed. Ecological dynamics is a multi-disciplinary framework that adopts concepts and tools of dynamical system theory, ecological psychology and complex system in neurobiology to investigate and model the relationships that emerge in extreme sports between athletes and their environment. In this relation, the performed behavior emerges through the continuous and active exploration of the environmental properties according to the individual intentions, body properties, perceptions and action capabilities.

In this narrative review, we present the framework of the ecological dynamics and how this theoretical framework would support a nonlinear pedagogy to skill acquisition and to design safe learning and training situations that are representative of extreme performance contexts. Second, we provide principles and examples to design intervention following nonlinear pedagogy, notably (i) to set a constraint-led approach (in particular task constraint), (ii) to implement conditions of practice (constant vs. variable, imposed vs. self-controlled), (iii) to promote adaptive and creative behavioral variability during practice.

## 2. An Ecological Dynamics Framework for Extreme Sport Understanding

The ecological dynamics theoretical framework is a multi-disciplinary framework based on the dynamical systems theory [1,2], ecological psychology [3] and a complex systems approach to neurobiology [4,5], and is supported by three main pillars [6,7,8]. The first pillar considers that movement coordination patterns are a dynamically functional relationship emerging from a set of interacting constraints, including the environment, the task and the resources of a performer [9]. Therefore, climbing performance should be analyzed on the ecological scale, which implies that the performer–environment coupling is the smallest unit of analysis to investigate climbing performance and expertise [10,11]. For example, in rock climbing, the rolling motion of the body is examined in reference to the surface of a climbing wall or cliff, rather than according to the longitudinal axis passing from the head to the feet of the climber, because this eco-physical variable would indicate whether the climber is facing the wall or is side on to the wall (for more details, see [12]).

Second, the ecological dynamics framework considers the performer–environment coupling as a complex adaptive system which means that the perceptual-motor behavioral organization exhibits *non-linear* and *non-proportional* properties [13]. This second pillar examines whether performance and skill acquisition improve linearly and proportionally with the increase in the constraints of the environment, the task and the performer [1]. The *non-proportionality* that exists between behavior and performance could be exemplified by sensitivity to initial conditions, in particular when during practice a small change in behavior can lead to a transition in performance and, conversely, switching between two behavioral patterns can lead to a marginal change in the performance outcome [14]. The *non-linear* relationship between behavior and performance can be observed in sudden transitions between two behavioral states, such as the change from walking to running on a treadmill, the task constraint increases linearly (the speed imposed by the treadmill rising incrementally), however, the movement changes in a non-linear manner [2]. Such non-linear behavioral changes could also be observed in climbing as climbers switched from a “face” body position to a “side” body position when the climbing holds orientation increases linearly from horizontal to vertical [12]. Based on previous findings [15], it can be postulated that a linear decrease in the depth holds would lead climbers to switch between grasping patterns. In the same vein, a linear increase in the steepness of a rock cliff from a positive inclination (i.e., ramp) to a negative inclination (i.e., overhang) would lead climbers to switch from biped to quadruped locomotion. Specifically, a particular ramp might favor smearing (i.e., using climbing shoe friction) whereas an overhang often involves actions such as arm pumping and feet hooking for improving one’s position on the surface.

Non-linearity could also relate to the presence of “multi-stability” [2,16] induced by the inherent degeneracy of perceptual-motor systems, suggesting that the behavioral structure can vary without compromising function in achieving the task/goal [4,5,17]. Multi-stability means that there is no one stable behavioral state to match a set of constraints in interaction; rather, there are multiple stable performance solutions that can emerge, depending on opportunities of action offered by the environment and perceived by the climbers according to their capabilities [3]. Finally, when interacting with the environment, climbers’ behavior dynamics exhibit periods of stability, destabilization and reorganization toward new coordination states that completely reshape the perceptual-motor repertoire [6]. Multi-stability in rock climbing could be observed in the large range of hand grasping patterns and body positions regularly used to grasp a hold [18,19]. In the same vein, multi-stability could be also observed in ice climbing, as climbers used several stable coordination patterns (e.g., horizontal-, diagonally-, vertical- and cross-located angular positions of the ice tools and crampons) and several types of actions (i.e., swinging, kicking or hooking) to anchor ice tools [20,21]. This multi-stability demonstrates the functional adaptation to dynamic environmental properties of the icefall, which changes in thickness, density and shape with air temperature, number of previous ascents (i.e., holes made by previous climbers), sun exposition, etc. For instance, the anchorage locations and the type of actions were selected to protect the icefall structure. Notably, climbers usually separate their ice tools from each other (e.g., by using diagonally and vertically angular positions between two ice tools) by 20 cm to protect icefall surface structure, which might be fragile in some parts. When the ice is dense without any holes, climbers usually swing their ice tools and kick their crampons. Conversely, when the ice is hollow and fragile, climbers hook holes with their ice tools and crampons. Thus, expertise in an extreme sport such as ice climbing could relate to the functional ability of the climber to exhibit multi-stability of coordination patterns to anchor ice tools in relevant locations and by using relevant types of action. This example is of particular interest because it suggests that minimizing risks in extreme sport could be achieved by switching between motor solutions without compromising function in achieving the task/goal. It highlights the importance of multi-stability as a mark of expertise, as an expert could use multi-stability as a “back-up plan”.

This second pillar suggests that learning and training in extreme sports would aim to safely explore new motor solutions in order to develop a larger motor repertoire (i.e., multi-stability). More than that, it invites us to reconsider the role of behavioral variability in skill acquisition. The ecological dynamics framework has highlighted that behavioral variability should not necessarily be considered as a deviation from expert behavior that should be corrected, nor as noise from an expert model that should be minimized to enable the production of a consistent, automatic and economic movement pattern. Instead, several studies (presented later in this narrative review) have provided evidence for the adaptive and functional role of movement and coordination variability in order to satisfy interacting constraints.

The third pillar suggests that coordination variability emerges from a continuous co-regulation of perceptual and motor processes, referred to as perception–action coupling. The use of information is founded on picking-up information for affordances that can “solicit” and “constrain” behaviors in a specific performance environment [3,22,23]. Considering an ecological scale of analysis, which implies the definition of an eco-physical variable to examine the relationships between an individual and an environment define affordances. Therefore, affordances are both objective and subjective to each performer since they are ecological properties of the environment picked up relative to an individual’s own action capabilities, i.e., they are body-scaled and action-scaled [24,25]. On one hand, body-scaled affordances relate to the relations between the body of the climber (such as height, limb sizes, hand area, which can influence the distance and shape of the hold that a climber can reach and grasp) and a relevant property in the environment. On the other hand, action-scaled affordances relate to how the climbers exploit their capabilities and how they behave relative to their environment [24]. For instance, Warren [26] emphasized that despite differences in body size, young adults accurately perceived stairs as no longer climbable in a bipedal fashion, when the step height exceeded 88% of their lower limb length. Regarding action-scaled affordances, other research investigated how individuals perceive maximal reach-and-grasp in rock climbing tasks [27,28]. The authors found that individuals with low levels of experience underestimated their maximal boundary of grasp, i.e., they miscalibrated their reach and grasp actions according to how far away they perceived the hold. More recently, in a reaching-and-grasp task, Seifert et al. [29] showed that advanced climbers had greater maximal action capability but did not act nearer to their maximal action capability than intermediate climbers. The authors suggested two possible reasons. First, because of their greater maximal action capability, advanced climbers perceive different opportunities for action (as they exploit degeneracy of perceptual-motor system and exhibit multi-stability) than intermediate climbers, notably in the case of more complex grasping tasks. Therefore, advanced climbers engage in different modes of action reflecting more effective chaining movements. Second, because in climbing the consequence of overestimation means falling and could cause injury or worse, climbers might safely scale their actions within their action boundaries to succeed and to prevent injury [30]. This could explain why both advanced and intermediate climbers scaled their action at the same ratio of their maximal action capability. Thus, skill acquisition in extreme sports would correspond to the improvement of perceptual accuracy toward better perceptual attunement and calibration according to maximal action capability, in order to functionally (re)organize and continuously regulate their motor behavior to achieve successful (i.e., safe) performance outcomes and to minimize risk-taking. Attunement relates to the pickup of more reliable information patterns in the energy arrays to guide action, while calibration concerns the appropriate scaling between information and an individual’s action capabilities [30,31]. Scaling to action capabilities allows the distinction between possible and impossible opportunities for action [24,32]. For instance, the wrong calibration might lead to “dyno” (i.e., dynamic move) whereas the edge of the hold is too small (such as a crimp) and not holly (such as a jug), which can be considered as a maladaptive behavior because it leads to failing to grasp and eventually to a fall. Such kind of wrong calibration is of great consequence in extreme climbing, such as “free soloing” (i.e., climbing without a rope), as any fall could cause death.

## 3. Nonlinear Pedagogy to Design Learning and Training in Extreme Sport: The Case of Climbing

### 3.1. Manipulating Constraints: Functional Exploration and Adaptability

Achieving a particular intended goal, such as reaching the top of a climbing route, requires exploiting the performance constraints through functional adaptation of movements. The set of constraints in which outdoor climbers perform are dynamically evolving over the course of their performance: surfaces, size and shape of the holds change along the route, fatigue in forearms rises as the climbing lasts, and weather conditions are liable to change. These dynamically evolving constraints from the environment and the organism invite continuous adaptation of climbers’ behavior [1]. Adaptability requires an appropriate balance between flexibility and stability in movements according to the performance context, that is, varying coordination patterns fitting in the set of constraints and maintaining a coordination pattern undergoing disturbances [33].

Functional adaptations to the performance context characterize skilled behaviors in climbing. For example, expert ice-climbers are able to demonstrate a larger range of inter-limb coordination patterns during performances in comparison to novices, who appear to be stuck into a smaller set of possible patterns [20,21]. Such difference in motor repertoire was also found in indoor climbing where skilled climbers were shown to be able to use various trunk orientations while climbing while novices rarely used alternatives to a face-to-the-wall trunk orientation [12]. These observed larger repertoire in skilled climbers support the exploitation of the performance constraints as they can demonstrate functional variability according to their intended goal and the performance context.

Adapted coordination patterns emerge from the performer–environment system. Interactions of the climber with the climbing route generate information specifying what are the possibilities of action (i.e., affordances [3]) that the environment offers. With practice, performers learn to pick up information more reliable for action and to scale this information to their action capabilities [24,34]. These learning processes are respectively called attunement and calibration and enable the accurate perception of affordances. Perception of affordances in the climbing environment was shown to be skill-level-dependent [35,36]. Indeed, when climbers were asked to recall the sequence of holds of climbing routes, expert climbers showed better performances than novices when recalling a difficult route. However, when the route was easy or impossible to climb, novice and expert performance did not differ in the recall task [35]. These results emphasize that affordance perception in climbing is specific to performers’ action capabilities. Using a similar recall task, another study asked both inexperienced and expert climbers to think aloud while recalling the route [36]. The results showed that while inexperienced participants focused on structural features of the holds (i.e., their size, shape, location on the wall…), the expert climbers recalled the possible actions they could perform with the holds or with a series of holds [36]. Thus, one challenge in climbing skill acquisition is to develop learners’ ability to perceive and act on climbing affordances, that is, perceives the climbing route not as a series of handholds with different shapes placed next to each other, but in a functional term as a possible chain of climbing actions.

Perception of affordances is an active process that relies on the performer’s exploratory activity [37]. Looking at the route, touching the surfaces of the holds or changing the trunk orientation are all ways to generate information while climbing [8]. More precisely, these actions enable the performer to pick up patterns of stimulation (e.g., optical, or mechanical) structured by the properties of the environment and his/her motion [38,39]. That way, performers can engage with their environment through different modes of exploration that support the specification of the affordances in their surroundings. For example, a study proposed to differentiate five climbing states according to hip and limbs movement or immobility: (i) looking at the route or resting (limbs and hip are immobile), (ii) adjusting the center of mass (limbs are immobile and hip is moving), (iii) determining which hold to use (position or orientation of the limb is changing), (iv) hold changing (moving limbs but not the hip) and (v) performing (the hip and at least one limb are moving simultaneously) [8]. These climbing states were assumed to participate to affordance perception on the climbing route by informing climbers about holds *reach-ability* (is the hold too far or can I reach it?), holds *grasp-ability* (is it a foot or a hand-hold? which grasping pattern should I perform?) and holds *use-ability* (what movement can I perform to exploit the holds and progress on the route?). 

However, the modes of exploration can be engaging and threatening to the climber’s safety. For example, exploration from a distance with the visual system is certainly safe whereas touching a handhold to try a grasping pattern or to get information about friction on a foothold implies that the limb used for exploration purposes is no longer used as support, which can be potentially threatening the postural stability of the climber [40]. In the literature about the development of locomotion, Kretch and Adolph [41] proposed the ramping-up hypothesis to capture how the modes of exploration are organized to specify affordances. This hypothesis argues that modes of exploration are organized in space and time so that performers progressively use more engaging modes. That is, the visual mode is usually the first one used as it can be performed from a distance. For example, occasional glances can be sufficient for humans to control locomotion on regular and safe terrain [42,43]. However, if the visual information is not sufficient to perceive what actions can be performed, then more engaging modes are used, such as touching the surfaces or testing alternative motor solutions [41,43]. This organization of exploration appears to fit what is observed in climbing. Notably, the number of exploratory hand movements was shown to decrease with practice [12,40] suggesting that as soon as climbing affordances can be perceived by other means, this engaging mode of exploration is avoided.

Additionally, the changes observed in climbers’ exploratory activity with practice are associated with attunement to the route affordances. If the number of exploratory hand movements decreases with practice, it is certainly because the holds *grasp-ability* and *use-ability* can be specified more reliably with visual information than they could during early practice [8]. Practice also affects the climber’s gaze behavior by decreasing the number of visual fixations performed and by reducing the randomness in the gaze path during climbs [40,44], supporting that climbers improved their ability to differentiate reliable optic information to guide their movements on the route.

These effects of climbing experience on exploratory activity are also supported by transversal studies comparing climbers with different skill levels. Indeed, more skilled climbers were shown to explore more with their hip than less skilled climbers who explore both at the hand and hip levels [45] and in ice-climbing, experts demonstrated more sensitivity to the environmental properties which helped them limit the number of ice tools and crampon actions they performed while climbing [21]. Such differences in exploratory activity are also due to the larger range of coordination patterns that skilled climbers can demonstrate. For example, as mentioned previously, the expert ice-climbers could limit their number of actions because they could both perceive opportunities for hooking holes in the ice and perform the inter-limb coordination patterns to exploit these opportunities [21]. Thus, conditions of practice should both guide learners’ attention toward relevant information for action and discover various coordination patterns to attain their intended goals.

One solution proposed to enhance climbers’ exploration of the available information and of new coordination patterns is to manipulate constraints in the practice environment to provide meta-stable conditions of performance. For example, handholds orientation was shown to directly affect the body postures [12]. A horizontal edge enables climbers to climb facing the wall (such as when climbing a ladder), whereas when the handholds edge is vertical, the climbers require climbing side to the wall to exploit the handholds properties. The latter pattern of trunk rolling motion is less common in beginners who reduce their adaptability to a route composed of handholds with a vertical edge, as shown by the greater number of exploratory and performative movements performed and the lower climbing fluency in comparison to how they perform on a route designed with handholds with a horizontal edge [12]. However, designing the same route with double edge handholds (i.e., handholds with both a vertical and a horizontal edge) provides a meta-stable condition that both enables novices the use of stable trunk rolling motion pattern, and to explore safely (i.e., to try a new pattern with the opportunity to draw back to former pattern) the side to the wall climbing pattern [12,45].

### 3.2. Implementation of the Conditions of Practice: Constant vs. Variable Practice, Imposed vs. Self-Controlled

Ecological dynamics emphasized the functional role of behavioral variability in learning in order to enhance skill transfer. The rationale is that exploration of various behavioral solutions during practice would support the development of the learners’ behavioral repertoire. The resulting broader behavioral tendencies would support a more cooperative relationship with new task dynamics, supporting specific or general transfer to a new performance environment [6,46]. Specific transfer reflects an appropriate fit between the learner’s intrinsic dynamics and the new task whereas general transfer expresses that the learner can further improve the fit with the new performance environment relying on the preexisting behavioral repertoire [46]. For example, the behavioral repertoire of indoor climbers was shown to support performance in ice climbing tasks, but this new activity (notably the tools manipulation and the exploitation of the environmental properties) required further practice for indoor climbers to functionally exploit the specific constraints and information–movement couplings of ice climbing, illustrating a general transfer of skill [21,46]. Thus, beyond increasing the number of coordination patterns discovered during practice, inducing behavioral variability aims to confront learners to various information–movement couplings. This would support learners’ attunement to more reliable information so that they could better specify the fit between the environmental properties and their action capabilities [47].

More broadly in the motor learning literature, externally induced variability in practice was originally hypothesized to improve learning (i.e., transfer and/or retention) in comparison to more repetitive practice protocol, such as constant practice or blocked schedule of practice, by supporting the optimization of a recall and a recognition schema [48] or by increasing the contextual interference between trials [49]. More recently, evidence advocated that the effects of external variability on learning may be more complex and would depend on the nature of the variations (for a review on the topic, see [50]). However, recent research highlighted some limitations of constant practice protocols in the context of the acquisition of complex perceptual-motor skills such as those required by climbers.

First, although the motor learning literature emphasized that constant practice protocol generates better performances on average during practice than variable practice conditions, it was also showed that the performance dynamics at an individual level could be quite different. For example, a constant learning protocol of a climbing task showed that individual learners could demonstrate three different dynamics of climbing fluency scores: (i) progressive improvement, (ii) sudden improvement characterized by initially stable performances and a later positive change in climbing fluency, and (iii) repetitive failure in task completion resulting in the absence of improvement in climbing fluency [51]. When authors looked at the behavioral repertoire of the learners, they could highlight that learners’ initial repertoire (here reflected by their tendencies to use different trunk rolling motions while climbing) was linked to the performance dynamics. More precisely, climbers able to vary between face-on and side-on postures could progressively improve their performances whereas those who were initially limited to face-on postures were required to discover alternative means to climb before improving their climbing fluency. However, climbers who could not discover these alternatives during practice kept on failing or showed a lack of improvement [51]. This relationship between an individual’s intrinsic dynamics and task dynamics highlights the need to provide variability in practice conditions to foster the discovery of new coordination patterns and to avoid failing learners to be maintained into unsuccessful performance environments.

Second, even when practicing an indoor climbing task, usually designed with different shapes of climbing holds offering various opportunities for action along the route, we showed that learning outcomes would be limited in terms of transfer to new climbing routes [40]. In this study, we showed that transfer of skill following such a constant practice protocol was limited to a new climbing route inviting low-order changes in climbing movements, i.e., the distances between handholds were increased so that more amplitude in climbing movements were required. The two other transfer routes were designed to either induce high-order changes in climbing actions (i.e., handholds were turned to invite new postures to use them) or no changes in climbing actions but the handholds shape was changed so that the grasping pattern would not change but would be less obvious. The results showed that changing environmental properties to design these two routes affected climbers’ performances by both deteriorating learners’ climbing fluency and increasing their visual search in comparison to the learning route on post-tests [40]. This highlights that the repetition of confrontations to a single learning environment restrained learners from developing perceptual-motor skills very specific to it, limiting their ability to overcome perturbations of their information–movement couplings in this performance context.

Thus, confronting learners with various performance environments may be an adequate solution to engage learners in the discovery of alternative movement solutions and to broaden the range of their experienced information–movement couplings with the aim of facilitating future confrontation to unknown performance environments [47]. Recently, we investigated the effect of both constant and variable practice protocol of a novel climbing task on the performance and visual activity of novice participants [52]. In this study, the constant practice condition consisted in repeating trials on a single climbing route whereas the variable practice condition involved that learners were confronted to both the same route as the constant practice group (but to a lesser extent) and to a new variant of this route on each session. Participants were tested on a transfer route and the learning route (i.e., the route on which both groups trained) at the start and the end of the protocols. As expected, the improvement in the climbing fluency scores on the learning route was higher for the constant practice group but interestingly, the results showed that the two practice conditions affected differently the learners’ gaze patterns on this route. First, participants in the constant practice group tended to keep their gaze on the handholds until their hand touched it whereas participants in the variable practice group were more proactive as their gaze shift occurred earlier in relation to hand contact. The gaze pattern of the variable practice group was also more proactive on the transfer route, whereas the constant practice did not change its gaze pattern between pre and post-transfer tests. Although the performance of the two groups was not different on the transfer test, these results suggest that variable practice conditions support the development of the generalizable exploratory activity. More specifically, here the change in the gaze pattern offered more time to the climbers to look for the next climbing actions, potentially facilitating the chaining of climbing actions in unknown routes. In contrast, the absence of transfer of the gaze pattern used by the constant practice group on the learning route suggests that although it may have participated in developing the better fluency scores on the learning route, it competed with the demands induced by the confrontation with a new environment. Thus, further research in this direction is needed to reveal the change in exploratory activity induced by variable practice conditions underlying positive transfer to a new performance environment.

Most of the variable practice protocol provides new practice conditions at a regular time interval for all participants in the intervention group. However, we preceded to highlight interindividual differences in learning dynamics. Moreover, a case study that used the same variable practice protocol as in [52] within additional post sessions phenomenological interviews, revealed that participants can also voluntarily limit their exploration of some climbing coordination patterns during practice if they feel unsafe when performing them [53]. Thus, although inducing external variability may “force” exploration, exploration in engaging activities such as climbing also requires the participants to intend to it. Developing a learning protocol more respectful of individual learning dynamics is a challenge that fully makes sense in engaging activities (i.e., extreme sports).

A proposed solution is to give learners the opportunity to choose when to be confronted with a new performance context. In the motor learning literature, such protocols were tested and the results showed that (i) learners adapted the task difficulty to their skill level, which resulted in a better success rate than those following a progressive and regular increase in task difficulty [54], (ii) learners tended to start practice with low levels of contextual interference and to increase this level at the end of practice [55] and (iii) learners showed better learning outcomes than those following imposed protocol [56,57,58]. The benefits of having control over the practice schedule were attributed to the autonomy-supportive learning environment in such condition [58,59], see also the OPTIMAL learning framework proposed in [60] and to the better fit between the learner’s progression and task difficulty [54,55], see also the Challenge Point hypothesis in [61]. Although these interventions appeared successful when learning laboratory tasks, we may expect that the level of engagement needed to perform in a new route may prevent some individuals to challenge them with novelty. In [52], a self-controlled variable practice group was also implemented. The results did not reveal any benefits in terms of performances on the learning route and the transfer route but, the participants in this group showed various trends in their gaze patterns, with some developing more proactive gaze patterns, such as those in the variable practice group, while others demonstrated gaze patterns closer to the constant practice group. These results suggest that some participants may have used the given control on their practice schedule to maintain themselves in a comfort zone rather than challenging themselves. However, we may expect that such protocol could also promote for others (notably more skilled participants) more active self-regulation during performance as learners are given the opportunity to engage with the design of their learning environment and they have the possibility to explore this environment [62]. In sum, the effects of self-controlled practice on individual learning dynamics may be a direction to further investigate in engaging activities such as (extreme) rock climbing, notably because it might (i) maintain a high level of attention, and (ii) help to focus attention on the most relevant information for action.

### 3.3. Conditions for Promoting Adaptive and Creative Behavioral Variability

Adapted and adaptive behaviors have recently been linked to creativity [63,64]. Interestingly in extreme environments, when optimizing a behavior that is already present in the repertoire to fit a new situation (i.e., to adapt) can be challenging, looking for and trying something totally new (i.e., to create) may even be more challenging but potentially necessary because of unknown and unexpected environmental conditions (e.g., changing weather, changing of ice properties). Commonly, creativity is understood to be the capacity to produce novel solutions to open-ended problems in daily life [65]. With reference to the cognitive perspective of creativity, divergent thinking allows an individual to create more alternative ideas [66], leading to a more diverse toolbox from which an individual can generate potential solutions [67,68], tying creativity to various adaptation processes [65,66]. As previously mentioned, any movement solution formed when an individual is exposed to a novel task would be the product of the characteristics of the individual, environment, and task [69], arising in temporal couplings between the three as the action unfolds. An individual capable of developing more movement solutions in response to a given situation is therefore termed more “flexible”. Flexibility forms the basis of adaptability, as the latter is one’s ability to respond effectively to a changing task, i.e., to achieve the outcome successfully (see Section 3.1). Creativity is therefore a form of flexibility when facing a situation for the first time.

Promoting a high level of creativity in the learning tasks, leading to high flexibility in the performers’ behavior is a potential key factor for learning and training for extreme environment sports. For instance, Komar et al. [70] recently showed that when introducing uncertainty in climbing an unknown route (i.e., decreasing the amount of available information by making visible only a few coming holds), the key feature of expert climbers is not only the ability to make relevant decisions but rather to keep the maximum number of options open as long as possible until they can make their decision on where to go next. More specifically, experts were organizing their feet positions to be able to reach the potential next holds on both left and right paths, waiting for the information on where to go to appear. As far as possible, experts will continue to perceive and keep open multiple solutions until they choose one (earlier shown in tennis [71]). Specifically, this ability allowed those experts to avoid going backward (e.g., going back on lower holds) to re-adapt their route and continue moving upwards. In extreme environments, such as mountaineering when going a few steps backward is not always possible, anticipating the possible routes and always keeping open multiple options necessitates being creative and flexible in order to be adaptive. More than introducing variability in practice, training for extreme sports may benefit from the infusion of uncertainty in practice because uncertainty will push performers to constantly develop and maintain alternative solutions. Probably more than any regulated sport, an extreme environment requires performers to face uncertain, unexpected and potentially novel situations, and coping with those appears key for performing. In that sense, motor originality (i.e., a unique solution a performer can create) seems to be a predictor of persistent behavior [64], helping to face the unexpected. An interesting observation from Richard et al. [64] also highlights that more creative and flexible performers perceive the task as less difficult than less creative and flexible people. This is of major importance as learners are keener to explore or challenge themselves when the task reaches an optimal level of difficulty. Playing with the information–movement coupling in designing learning environments [13], specifically with how much information is available, can be a key pedagogical component to prepare learners for extreme environments.

## 4. Conclusions

In this narrative review, we conceptualized how, within the framework of the ecological dynamic, environments, conditions of practice and engagement would challenge the acquisition of perceptual-motor skills in extreme sports, such as climbing. In particular, skill acquisition should be investigated at the ecological scale, meaning that extreme sports practitioners should consider the coupling between performers and their environment (involving mutuality and reciprocity) in studying how they behave when facing a set of constraints in interaction. For this purpose, the eco-physical variable should be investigated and would allow tracking the behavioral dynamics to better understand emergent movement and performance variability as exploitation of degeneracy in the perceptual-motor system. We suggest that this behavioral variability could also be induced via manipulating constraints so that multiple affordances can be functional. This allows the individual to explore available system degeneracy by harnessing self-organizing processes. The challenge for the extreme sports practitioner is how to set up variable practice and uncertainty for efficient exploration and creativity in a manner that avoids dangers and injuries. Representing or “*sampling”* uncertainty within the relative safety of indoor or dry-tooling settings may be one intervention for preparing climbers for performance in extreme environments, with respect to the constraints manipulation, the nature and level of uncertainty that they represent in the learning design. For instance, uncertainty could be promoted in teaching and induced by manipulating (i) visual information during training, such as preventing route preview or requesting climbers to switch from one route to another route, in order to find backup strategies, and (ii) haptic information during training on indoor routes, such as changing the shape, texture, size and distance between holds to hide relevant information in order to further develop perceptual attunement and calibration, i.e., to develop functional exploration and creativity.
